# The Impact of Mindset on Self-Tracking Experience

**DOI:** 10.3389/fdgth.2021.676742

**Published:** 2021-08-03

**Authors:** Elçin Hancı, Peter A. M. Ruijten, Joyca Lacroix, Wijnand A. IJsselsteijn

**Affiliations:** ^1^Department of Industrial Engineering & Innovation Sciences, Eindhoven University of Technology, Eindhoven, Netherlands; ^2^Philips Research, Eindhoven, Netherlands

**Keywords:** mindset theory, self-tracking, self-compassion, goal orientation, motivation

## Abstract

Self-tracking technologies aim to offer a better understanding of ourselves through data, create self-awareness, and facilitate healthy behavior change. Despite such promising objectives, very little is known about whether the implicit beliefs users may have about the changeability of their own behavior influence the way they experience self-tracking. These implicit beliefs about the permanence of the abilities are called mindsets; someone with a fixed mindset typically perceives human qualities (e.g., intelligence) as fixed, while someone with a growth mindset perceives them as amenable to change and improvement through learning. This paper investigates the concept of mindset in the context of self-tracking and uses online survey data from individuals wearing a self-tracking device (*n* = 290) to explore the ways in which users with different mindsets experience self-tracking. A combination of qualitative and quantitative approaches indicates that implicit beliefs about the changeability of behavior influence the extent to which users are self-determined toward self-tracking use. Moreover, differences were found in how users perceive and respond to failure, and how self-judgmental vs. self-compassionate they are toward their own mistakes. Overall, considering that how users respond to the self-tracking data is one of the core dimensions of self-tracking, our results suggest that mindset is one of the important determinants in shaping the self-tracking experience. This paper concludes by presenting design considerations and directions for future research.

## Introduction

Self-tracking tools collect behavioral and bodily data and provide feedback to the user. By increasing self-awareness and providing motivational incentives, self-tracking devices aim to facilitate positive health-related behavior change (1, 2). Despite the rising popularity of self-tracking tools and practices, very little is known about whether and to what extent the implicit beliefs users hold about the changeability of their own behavior influence how they evaluate and respond to self-tracking data. Such implicit beliefs, or mindsets, refer to the attitudes an individual holds in regard to the extent to which human qualities or abilities are malleable (3). Individuals with a *fixed mindset* perceive human abilities, such as intelligence, as stable traits that are more or less fixed for any given person. People with a *growth mindset*, on the other hand, perceive their abilities as amenable to change, with the potential for improvement through learning and experience (3). It is important to note that the mindset theory has been challenged in view of the replication crisis in psychology over the last decade. Yet, the findings from recent studies, including those of Yeager et al. (4), who performed a preregistered study, and a meta-analysis conducted by Burnette et al. (5) have shown it to have sufficient ground to study further. In their conclusion, they write: “The present meta-analysis suggests that mindsets matter. That is, implicit theories are indeed consequential for self-regulatory processes and goal achievement. (…). One important conclusion from the present meta-analysis is that the associations of implicit theories with self-regulation are not straightforward and that perhaps the literature would be better served by asking when and how implicit theories are consequential for self-regulation rather than asking if incremental theories are generally beneficial.” The present paper seeks to explore whether an incremental implicit theory, or growth mindset, is specifically beneficial when people are engaged in technology-assisted self-regulatory behaviors, or, phrased differently, in self-tracking.

Different beliefs about the underlying nature of ability are found to influence how individuals interpret and respond to notions such as effort and failure (6, 7). People with a growth mindset believe that abilities can be learned and improved with effort, negative feedback, setbacks, or failure to reach a goal are more likely to be interpreted as opportunities for learning, and they are likely to adjust their efforts to realize those learnings (8). People with a fixed mindset, on the other hand, implicitly expect their abilities to evolve independently from the amount of effort invested, and therefore setbacks are taken as failures and personal shortcomings of their competence and potential. People with a fixed mindset are more likely to take failure personally (9). This is so deeply ingrained that neuroscientific research shows that people with a fixed mindset demonstrate more emotionally significant responses toward a negative performance feedback than those with a growth mindset (10).

By means of monitoring personal data and providing ubiquitous feedback on various aspects of daily life, self-tracking practice becomes not only a mere repository of highly personal information but also a representation of the self in a datafied fashion. The ways in which digital self-tracked data generate users' “data selves” have been widely studied in ethnographic and sociological work with the concept of “data self” appearing under different names such as *digital twin, data double*, or *digital doppelganger* (11–13). The underlying idea is that “accumulated self-tracked data creates an entity within self-tracking systems that reflects, engages with, and mimics the user” (13). As a consequence of this, the way individuals engage with themselves mirrors the way they engage with the self-tracked data. Given the profound role, mindsets play in revealing a person's view of the self, we propose that it is likely that mindsets continue to be determinants in interpreting one's self-tracked data. Thus, a better understanding of users with different mindsets becomes highly relevant in determining the experiences that self-tracking offers as well as the potential for sustained, long-term self-tracking.

In the following sections, we introduce the theoretical foundations concerning the growth and fixed mindsets, and extend what growth and fixed mindsets mean in the context of self-tracking, in particular in relation to its behavioral and motivational implications. This provides the basis for our examination of how these mindsets relate to experiences with self-tracking, including the varying levels of self-compassion users have toward themselves.

### Mindsets: Growth vs. Fixed

Research shows that mindsets can change over time with new experiences and targeted interventions. This flexibility of mindsets can be particularly interesting because they are domain specific, meaning that someone can hold a fixed mindset in one area (e.g., exercise) and a growth mindset in another one (e.g., academic success). Learning how to adopt a growth mindset in various areas can be helpful in providing continuous opportunities for growth. In their intervention study, for example, Aranson et al. (14) showed that when students were told that intelligence is something that can be improved (growth mindset intervention), they received higher grades compared to when they were told that intelligence is an inborn ability (fixed mindset intervention). Intellectual performance, in fact, was the primary domain of interest for the emergence of the mindset theory. Later on, scholars have extended research on the impact of implicit theories to other areas of personal achievement, including parenting, athletics, leadership, as well as fitness, and health (3, 15–17). For example, stimulating people in a growth mindset can be an effective intervention to prevent weight gain following dieting setbacks (18).

One of the domains in which mindsets play an important role in understanding is exercise. The difference in the belief of whether abilities can be improved or not affects how one approaches exercise as a concept. When an athlete, be it professional or an amateur, holds a growth mindset, training becomes a platform to display one's willingness to fail for the sake of improvement and therefore possible setbacks are perceived as opportunities that are embraced rather than as something to be terrified of and avoided completely. Children who believe that athletic ability is open to improvement are more likely to attend and enjoy physical education (19). The belief about the malleability of personal qualities does not only shape the understanding of exercise as a concept but also the sport performance itself. In a coach–athlete relationship, for example, behaviors and feedback of the coach in response to an athlete's skill performance were found to affect that athlete's perceived competence and resilience; when the coach provides feedback that is in line with a growth mindset, athletes are more likely to believe that they can perform better, which indeed leads to better performance (20).

#### Mindset and Achievement Goal Theory

The difference in the belief of whether abilities can be improved or whether they remain stable has been found to inspire different goal orientations. Achievement goal theory (AGT) is a 2 × 2 conceptualization that differentiates the type of goals based on how competence is defined (mastery vs. performance) and valenced (approach vs. avoidance) (21, 22). Four different types of goals are: mastery-approach (focuses on intrapersonal competence), performance-approach (focuses on normative competence), mastery-avoidance (focuses on intrapersonal incompetence), and performance-avoidance (focuses on normative incompetence) (see Table 1 for examples).

**Table 1:** A 2 is × 2 achievement goal framework.

	**Approach orientation**	**Avoidance orientation**
Mastery goal orientation	I want to improve my stamina by working out regularly	I am concerned that I may not run as regular as I used to as I am getting older
Performance goal orientation	It is important for me to run faster than my friends do	My goal is to avoid performing poorly in my running group

Mindsets are considered to be the dispositional variables *that are predictors of achievement goals* (22). For someone with a growth mindset where abilities are believed to be malleable and effort and commitment are valued, people are likely to have mastery goals (21). They also seem to adopt a more approach orientation whereby challenges are sought to be successful. For people with a fixed mindset where abilities are believed to be inborn, they are likely to pursue performance goals where the main focus is demonstrating the adequacy of the ability. They are also more likely to adopt avoidance motives as the outcomes are considered to determine one's self-worth and any failure leads to a decline in self-worth.

#### Mindset and Motivation

Mindset theory posits that individuals' beliefs about malleability of their abilities affect the level at which one exhibits self-determination. Self-determination theory (SDT) states that a person's motivation to perform a behavior vary in the extent to which their regulation is autonomous and makes a distinction between the performing activities that are rewarding in and of itself, which is called intrinsic motivation, and ones where the performance is determined by the external factors such as rewards, deadlines, etc., which is known as extrinsic motivation (23). SDT positions extrinsic motivation on a continuum scale and states that the more internalized the regulation of the behavior, the less contingent it is to external factors. Four different types of extrinsic motivation are distinguished depending on the level of internalization of the behavioral regulation people experience, which are external motivation, introjected regulation, identified regulation, and integrated regulation. Intrinsic, integrated, and identified regulation together form a subcategory of motivation called autonomous or internal motivation (24). When a person has high levels of intrinsic, integrated, or identified regulation, their motivation for engaging in a certain behavior is voluntary and without a lot of external pressure (25). For individuals that are autonomously motivated, higher levels of concentration, better performance, and higher levels of persistence were reported (26). Introjected and external regulation form a subcategory of controlled or external motivation (24). When a person has a high level of controlled motivation, it is likely that they engage in activity tracking because of external influences like gaining rewards or being pushed. It has been shown that implicit beliefs people hold affect their motivation when performing a task. For example, students who hold a fixed mindset about intelligence were found to have lower intrinsic motivation toward schoolwork than those with a growth mindset (27). It was because for students who think intelligence is a fixed trait, schoolwork was perceived as something to validate their skills rather than as a platform to learn and grow.

### Self-Compassion

Overall, the belief that qualities are carved in stone or that they can be cultivated seems to have an overarching influence on one's daily life by means of regulating various psychological processes; mindsets change the meaning of effort and failure and influence individuals' achievement goal orientations and self-determination. Given that the level of perseverance and resilience changes based on different mindsets, one might expect that mindsets also correspondingly affect how forgiving and tolerant people become toward their own inadequacies and failures, also known as self-compassion.

Self-compassion involves being understanding and kind to oneself when confronted with personal shortcomings and failures. Neff (28) proposes that self-compassion is comprised of three dimensions with negative and positive poles on each side: self-kindness vs. self-judgment, common humanity vs. isolation, and mindfulness vs. over-identification. Self-kindness involves extending kindness and being gentle with oneself when confronted with a setback, whereas the opposite is self-judgment, which involves suffering in the form of frustration and self-criticism. Common humanity is about recognizing one's personal inadequacies as part of a larger and shared human experience, whereas the contrary is called isolation where seeing failure as something unique to the individual alone. Lastly, mindfulness involves being acceptant and non-judgmental toward thoughts and feelings, instead of identifying with thoughts and feelings.

Literature indeed shows that the level of self-compassion differs depending on the implicit beliefs one holds. Believing that qualities can be cultivated, those with a growth mindset tend to express higher levels of self-compassion and are more likely to be tolerant to their mistakes as these are considered as learning opportunities (29). Believing that abilities can be improved encourages individuals to seek for accurate information about themselves even if it is not flattering. Whereas, people who hold a fixed mindset and believe that abilities are inborn and cannot be changed or improved, it is likely that they will be swept away by the negative reactivity. For someone with a fixed mindset, therefore, “*a failure becomes no longer an action (I fail) but an identity (I am failure)* [3, p 34].”

### Mindset in Self-Tracking Practice

When adopting a healthier lifestyle using a self-tracking technology, these psychological processes that are discussed become highly relevant. To adopt a healthier habit, one must set goals, be able to cope with setbacks when not reaching the goals, continue to be consistent in tracking despite obstacles, incorporate the self-trackers' feedback and be kind to herself simultaneously to be able to continue to be encouraged. However, to be able to self-regulate this successfully, first, one should believe that changing behaviors and forming new habits *via* the use of self-tracking is possible. This belief will promote confidence in one's behavior change capacity and enhance their interest to be engaged with self-tracking technology.

As mindsets are domain specific, it is possible that users hold different mindsets in the context of self-tracking. A user with a fixed mindset would believe that changing one's behaviors using self-tracking is unlikely while a user with a growth mindset would hold the belief that behaviors are malleable and self-tracking practice can help to pursue a healthier lifestyle. Given that self-tracking technology makes health-related lifestyle features subject to measurement, it is likely that users with different mindsets respond to such normative evaluations differently, interpret the meaning and self-relevance of the numbers in different ways, and correspondingly vary in their duration of self-tracking use.

Mindsets become relevant in understanding the way self-tracking data is interpreted. As fixed and growth mindsets differ in the way the meaning of effort and failure are translated, it is possible that the accumulated data are interpreted and shared differently depending on user mindsets. Those with a fixed mindset have the urge to document their supposedly fixed traits and the accumulated data are there to prove these static abilities. Any mismatch between what the data shows and what is expected from it can make individuals judgmental toward their own performance. Correspondingly, they can also be more reluctant to share their “failures” online and stick merely to the information that would make them look more “accomplished.” Users with a growth mindset, on the other hand, deploy the monitored data to reflect on their mistakes and cultivate from there. Not reaching a goal can still be painful but can also be seen as something to learn from. Compared to the users with a fixed mindset, those who hold a growth mindset can be more honest and open when it comes to sharing the self-tracking goals they could meet and provide a less biased impression of themselves.

It is also possible that mindsets play a role in understanding the reasons for engaging with self-tracking. When validation of the self becomes the primary concern for the fixed mindset, it is possible that the reasons to engage in self-tracking become less self-determined and more externally oriented. The main focus for fixed mindset people, therefore, seems to be the judgment of others, which corresponds to an externally driven type of motivation. When believing that success is about learning, users with a growth mindset treat data no longer as means of validation but instead as a material to reflect on in order to grow. The role attributed to the self-tracking practice, hence, becomes more self-determined. Having a growth mindset would allow users to enjoy the activity of self-tracking in and of itself, regardless of any external rewards, corresponding to an internally driven type of motivation.

Overall, we propose that mindset in self-tracking will have a multilayered influence on self-tracking use. First, it will affect users' goal setting orientations; while users with both fixed and growth mindsets encompass the desire to improve, the reasons to do so can differ based on users' mindsets. When setting a self-tracking goal, a user's mindset will also play a role in the interpretation of the feedback. For those with a fixed mindset reaching a goal can be interpreted as an affirmation of talent and determining self-worth, while not reaching a goal becomes a personal failure that is to be avoided and not to be shared. Finally, mindsets may also influence psychological coping mechanisms users apply; in case of a setback, those with a growth mindset will be more likely to show more self-compassion than those with a fixed mindset.

### Current Study

In this study, we sought to investigate the interplay between mindset, motivation, and self-compassion in the context of self-tracking. In order to test how user's mindset about behavior change with the use of self-tracking may relate to their self-compassion and motivation to self-track, we conducted an online survey. First, an open question was used to uncover people's feelings and attitudes when their self-tracking goal was not met, to help us indicate the type of mindset these users have. This qualitative approach helped us to delve deep into how people with different mindsets experience self-tracking. We then tested quantitatively if users with different mindsets also differed in their motivation to self-track and self-compassion, as shown in the literature albeit in different areas. We expected that users with a growth mindset would be more autonomously motivated toward the use of self-tracking and more self-compassionate toward themselves than those with a fixed mindset.

## Method

### Participants and Design

Participants were recruited *via* Amazon mTurk. An *a priori* power analysis indicated that with a sample of 300 participants, we would have 90% power to be able to find effects of mindset on the two types of motivation (with an alpha level of 0.025) as small as a Cohen's *d* of 0.41. The same sample size would allow us to find correlations as small as *r* = 0.18. After leaving out the outliers and participants who failed to pass an attention check, the sample for the final analysis was composed of 290 participants (118 female) ranging in the age from 18 to 69 (*M* = 32.5, SD = 8.7). Participants met the inclusion criteria of (1) owning a self-tracking tool and (2) tracking at least one health-related lifestyle behavior (e.g., steps) using their self-tracker. The most frequently tracked feature was exercise (e.g., number of steps taken), followed by sleep and calorie intake. The survey consisted of three questionnaires measuring users' mindset about behavior change with the use of self-tracking, their motivation to self-track and self-compassion as a personality trait. Alongside of survey items, participants were also given an open-ended question, and asked to reflect on a recent self-tracking goal they set for themselves but failed to achieve. Completing the survey took about 10 min for which participants received a compensation of US$2.

### Measures

The survey consisted of both a qualitative measure (i.e., an open question) and three questionnaires, which were adjusted accordingly. The self-compassion and mindset questionnaires were answered on a seven-point Likert agreement scale (1 = strongly disagree and 7 = strongly agree) and motivation questionnaire on a seven-point correspondence scale (1 = does not correspond at all and 7 = corresponds exactly). The order of the questionnaires was displayed in a random order.

#### Mindset

To measure users' mindset about behavior change using self-tracking technology, the eight-item self-theory version of the implicit theories of intelligence scale was used and adopted to fit into the self-tracking context (original item *three. To be honest, I don't think I can really change how intelligent I am* is modified to *three. To be honest, I don't think I can really change my behavior with a self-tracking device*) (30). The newly adopted scale demonstrated low inter-item reliability (Cronbach's α = 0.48) with items three and four having the lowest mean scores. Furthermore, the scale with eight items has been subjected to principal component analysis (PCA). This analysis showed that items three and four did not fit any of the two components (see Table 2A). When we removed those two items from the analysis, again two components showed. However, as can be seen in Table 2B, all items also load high on the first component. After removing items three and four, the inter-item reliability increased to Cronbach's α = 0.66. We therefore decided to average the remaining six items into one score for mindset, where higher scores on this measure corresponded to growth mindset and lower scores corresponded to a fixed mindset (see Appendix A for an overview of all items). We reflect the potential reason for the second component being shown up in the analysis in section Discussion.

**Table 2A:** Component matrix with all mindset scale items included.

	**Component**	
**Items**	**1**	**2**
Recoded_mindset1	0.534	0.705
Recoded_mindset2	0.439	0.762
**Recoded_mindset3**	**−0.666**	**0.259**
**Recoded_mindset4**	**−0.131**	**0.563**
Mindset5	0.732	−0.092
Mindset6	0.694	−0.235
Mindset7	0.540	−0.274
Mindset8	0.669	−0.130

*Bold values represent factor loadings for the items that diverge from both components*.

**Table 2B:** Component matrix with items three and four of the mindset scale excluded.

	**Component**	
**Items**	**1**	**2**
Recoded_mindset1	0.628	0.660
Recoded_mindset2	0.568	0.708
Mindset5	0.715	−0.187
Mindset6	0.645	−0.378
Mindset7	0.524	−0.453
Mindset8	0.673	−0.299

For the analysis, we have split the mindset score up in line with our hypothesis to create fixed vs. growth mindset binary groups. This procedure is explained in detail in section Results.

#### Self-Compassion

For the assessment of self-compassion as a personality trait, the 12-item self-compassion scale-short form (SCS-SF) was used (31) (Cronbach's α = 0.73) (see Appendix B). The scale measures all six facets of self-compassion as covered by the literature, which are self-kindness, self-judgment, common humanity, isolation, mindfulness, and over-identification (28). Items from 7 to 12 were reverse coded; higher scores meant participants were more self-compassionate toward themselves and more tolerant toward their mistakes while those with lower scores indicated lower levels of self-compassion and being rather judgmental toward their mistakes and less at peace with themselves. Even though the scale allows us to measure all six facets of self-compassion separately, this is outside of the scope of the current manuscript. We regarded and addressed self-compassion as one variable.

#### Motivation

In order to measure to what extent users were self-determined to using self-tracking, the Revisited Sport Motivation Scale (SMS II) was used and adopted to fit into the self-tracking context (25) (see Appendix C). The SMS II is a well-established measurement tool and measures all types of motivations as covered by SDT and thus helps to determine not only the quantity but also the quality of motivation. The original scale consisted of 18 items with 3 items per type of motivation (intrinsic, identified, integrated, and introjected) and 3 items for amotivation. The three items measuring amotivation were inapplicable and thus removed as our target population consisted of people who are currently self-tracking, indicating that they should at least have some level of motivation for wearing it. Moreover, one item with the lowest alpha level for each type of motivation was left out to decrease the length of the scale while maintaining the validity. Some items of the scale were modified to make them applicable to the self-tracking context (e.g., original item *because practicing sports reflects the essence of whom I am* was modified to *because self-tracking reflects the* essence *of whom I am*). In the end, 10 items remained for the motivation scale and 2 subscales were calculated depending on the degree to which the motivation is self-determined. In line with Deci and Ryan's (23) categorization of the degree to which motivational bases are self-determined, six items measured intrinsic, integrated, and identified motivation and were grouped as autonomous motivation (Cronbach's α = 0.79). The remaining four items measured introjected and extrinsic motivation and were grouped as controlled motivation (Cronbach's α = 0.76).

#### Experiences With Self-Tracking

Alongside the survey items, participants were also given an open-ended question and asked to reflect on a recent self-tracking goal they set for themselves but could not manage to reach within their (time/form) restrictions. Setting a goal is a common practice among self-tracking users and it can be done for various purposes; a goal can be something to track something specific and performance focused (e.g., to run 8 km without stopping) or something that is broader (e.g., be physically active) and set to improve one's skills (e.g., walk more than the previous day for a month). Not reaching these goals can be contingent upon different reasons, which could or could not be controlled (e.g., bad weather vs. feeling lazy). Therefore, the type of goals users set for themselves and the ways in which they respond to their setbacks can be insightful for a better understanding of how users experience self-tracking. We let participants express their feelings that elicited from not reaching their goals and by doing so, we aimed to get a deeper insight about the differences user mindsets might bring in experiencing self-tracking.

## Results

### Quantitative Analysis

We first quantitatively examined the role of mindsets in relation to other relevant variables, namely motivation and self-compassion as a personality trait. The interplay between these variables has been previously investigated in different areas but particularly not in a self-tracking context. Accordingly, we tested two hypotheses; (H1) we expected that users with a growth mindset would be more autonomously motivated toward the use of self-tracking than those with a fixed mindset and (H2) that people with a growth mindset would be more self-compassionate toward themselves when faced with a disappointing performance.

Mindset measurement as operationalized through the six-item mindset questionnaire was a continuous variable, where higher scores indicated a growth mindset and lower scores indicated a fixed mindset. To arrive at a fixed vs. growth mindset dichotomy, we split our complete sample based on *z*-scores of the mindset measurement with a *z*-score = 0 as a cut-off point, creating two groups: people with a positive *z*-score are grouped as growth mindset (*n* = 120) and people with a negative *z*-score are grouped as fixed mindset (*n* = 170). A Shapiro–Wilk test on the mindset variable showed that the scores were not normally distributed, *W* (290) = 0.95, *p* < 0.01. To compare autonomous and controlled motivation scores across the users with different mindsets, a Mann–Whitney *U* test, a non-parametric equivalence of independent *t*-test, was conducted. The results showed that having a fixed vs. growth mindset had an influence on the extent to which users report self-regulated motivation with regard to self-tracking; those with a fixed mindset were more likely to have controlled motivation for self-tracking compared to those with a growth mindset (Mann–Whitney *U* = 3,945.5, *z* = −8.91, *p* < 0.001) while mindset type did not matter for autonomous motivation (Mann–Whitney *U* = 9,568, *z* = −0.9, *p* > 0.05).

To test if self-compassion as a personality trait differed across different mindsets, we first used mindset measurement as a continuous variable and showed that the higher people scored on the mindset scale, the more self-compassionate they were [*r* (288) = 0.31, *p* < 0.001]. Additionally, we have also explored the relation between the duration of self-tracking use and the mindsets users hold. The results showed that there was a positive correlation between mindset and the longevity of self-tracking use; the higher participants scored on growth mindset, the longer they had been practicing self-tracking [*r* (288) = 0.25, *p* < 0.01].

### Qualitative Analysis

To gain a better understanding of the experiences users had when faced with a setback, answers from the open-ended question were analyzed by conducting a deductive content analysis. Different from a thematic analysis where the researcher comes up with the codes that are essence-capturing, in the content analysis, codes are developed *a priori*. The purpose of the content analysis is to derive insights from the responses by means of identifying the patterns *via* predetermined coding schemes and transforming these observations of patterns into quantitative data. As we have used self-compassion and goal achievement theories as theoretical lenses for our analysis, the codes developed *a priori* were based on six facets of self-compassion (self-kindness vs. self-judgment, common humanity vs. isolation, and mindfulness vs. over-identification) and types of goals (mastery vs. performance).

As the analysis was based on the differences in frequency of coding across the two mindsets, it was important to have equal numbers of responses from both fixed and growth mindset users so that the differences in the frequencies are attributed to the mindsets themselves. Furthermore, since the concept of mindsets has not been studied in self-tracking context before, we argue that it is reasonable to scrutinize the extreme ends of the mindset continuum and investigate if people who fall into the highest vs. lowest scores on the mindset scale show any difference. In order to do so, 50 participants who scored the highest and 50 participants who scored the lowest on the mindset scale were chosen, leading to a data set of 100 responses in total.

For the analysis, we were mainly interested in two questions: (1) Is there a difference in how participants in fixed vs. growth mindset express self-compassion after getting reminded of their failures? and (2) Is there a difference in the type of goals they set for themselves? Coding schemes for each question were prepared prior to the analysis. For the first question, the coding scheme consisted of six elements of self-compassion, which are self-kindness vs. self-judgment, common humanity vs. isolation, and mindfulness vs. over-identification (28). The coding scheme for the second question consisted of two different types of goals; mastery goals, which focus on competence and improvement and performance goals, which focus on the outcome. We expected users with a growth mindset to be more likely to express self-compassion in their responses when reflecting how they dealt with not managing to achieve their self-tracking goals compared to those with a fixed mindset. We also expected that those with a growth (vs. fixed) mindset would be more likely to set mastery (vs. performance) goals.

In order to avoid any possible bias, the coder was not aware of the mindset score of the participants until the coding was completed. Inter-coder reliability of the content analysis was tested by an independent coder; there was 86% agreement and disagreements were resolved upon discussion.

#### Mindset and Goal Orientation

**Figure 1:**
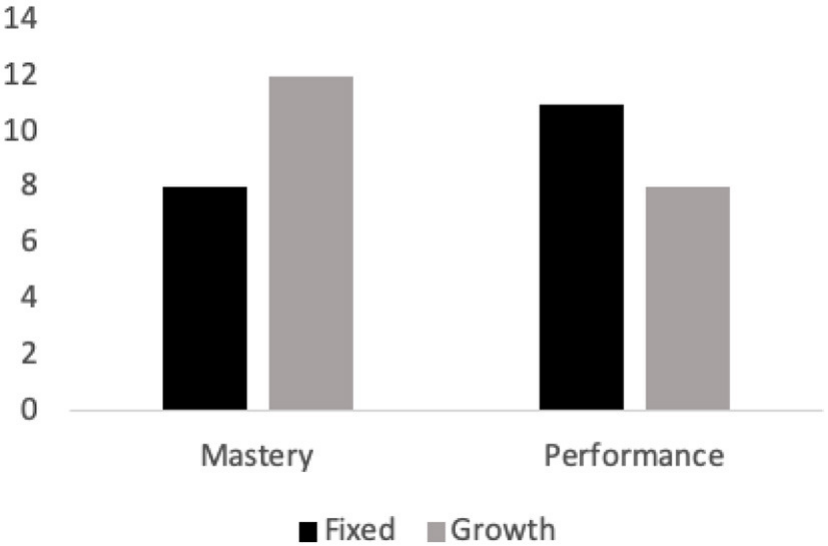
Frequency of mastery vs. performance goal orientations across participants with fixed vs. growth mindset.

Literature shows that the mindsets individuals hold and their goal orientation often goes hand in hand; those with a growth mindset are likely to pursue mastery-oriented goals as they mostly focus on increasing their abilities over time while those with a fixed mindset are likely to pursue performance-oriented goals where the main concern is to document and measure the adequacy of their abilities. In this study, we were interested if this holds also in self-tracking context. Findings of the analysis showed that, in line with the literature, a majority of the self-tracking goals that were set but yet not reached among users with a growth mindset involved mastery-oriented goals [e.g., …*working out daily* (p. 319), …*to increase my steps* (p. 13), …*to increase my gym attendance* (p. 321)], see Figure 1. While not everyone in the selected sample explicitly mentioned their goals, among the ones who did, those with a growth mindset were almost twice likely to set mastery goals compared to those with a fixed mindset; they were mostly concerned about using self-tracking tools to set goals that can aid in forming healthier habits and develop their competence. Users holding a fixed mindset, on the other hand, more often aimed for performance goals where the pursuit of achievement is determined by a focus on the measured outcome [e.g., …*lose 5 pounds in a month* (p. 170), *My goal was to lose all my baby weight…*(p. 212)]. Although elements of mastery and performance goals were found across all the users, for users with a fixed mindset, self-tracking goals were more frequently framed as a demonstration of competence, rather than as a process of development.

**Figure 2:**
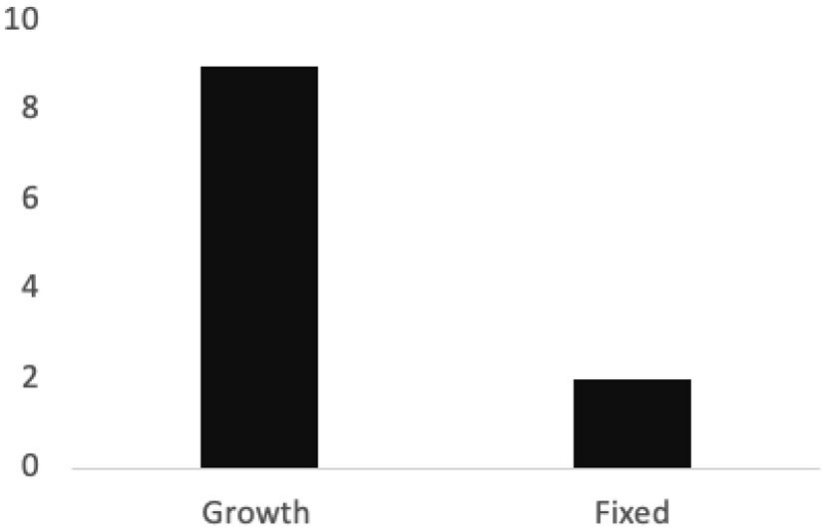
Frequency of resilience statements across participants with growth vs. fixed mindset.

Findings also showed that users' resilience as a response to their failures has aligned with their type of goal orientations (see Figure 2). Those users with a growth mindset who tend to set mastery goals were also more persistent in their efforts. They tended to think of ways of overcoming the obstacles that caused them to fail reaching their goal […*I thought of ways to reach that goal if the weather had been bad again* (p. 132), *When I am in a better financial situation I want to use a running coach* (p. 159)], and persistent in their will to keep trying [*I am determined to train back up to that point again* (p. 203), *I knew that I'd eventually get back to trying to reach my goal*… (p. 286)]. In the face of failure, they did not take it as a discouragement and continued to improve themselves [*I won't let it stop me from continuing to eat healthy and exercise during and after my trip* (p. 32)]. In comparison to those with a growth mindset, responses from users with a fixed mindset were less likely to report setbacks with a resilient attitude. As a matter of fact, the majority of the resilient statements came from those with a growth mindset. Users with a fixed mindset, on the contrary, were sometimes even likely to report setbacks with a withdrawal of effort [e.g., *I ended up giving up the goal…* (p. 304)]. When setting a goal with improvement in mind, self-tracking users became more likely to show resilience and continue their attempts toward reaching their goals.

#### Mindset and Self-Compassion

**Figure 3:**
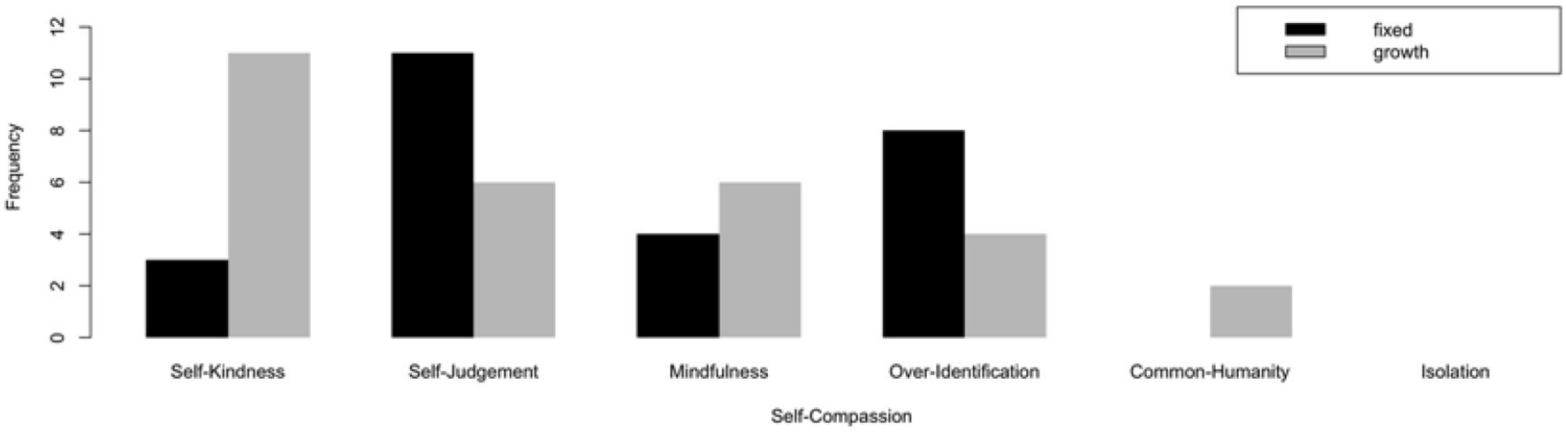
Frequency of self-compassion dimensions across participants with growth vs. fixed mindset.

By setting goals with the aim of learning and growing in mind, people with a growth mindset tended to be less afraid of failures and making mistakes, and therefore, they respond to setbacks with more self-compassion. By asking users directly to reflect on their recent self-tracking goal that they could not meet, we aimed to explore if users with a growth mindset respond to their setbacks with a more positive attitude. For the analysis, we examined the three dimensions of self-compassion with their positive and negative poles, representing all the six components: self-kindness vs. self-judgment, mindfulness vs. over-identification, and common humanity vs. isolation. The frequencies with which each of these dimensions was mentioned are shown in Figure 3.

##### Self-Kindness-vs.-Self-Judgment

The results of the quantitative content analysis showed that the contrast between mindsets was most evident in the self-kindness vs. self-judgment facet of self-compassion. In line with our expectations, after being reminded of their setbacks, participants with a growth mindset were thrice more likely to express self-kindness than those with a fixed mindset (see Figure 3). They reflected friendlier [“*I was happy with the goal I reached* …” (p. 121)] and more supportive attitude toward themselves [“*…Overall I did great”* (p. 32)]. Focusing on improvement rather than the outcome seemed to make them more appreciative of the effort that is put forward [“*…I was proud of having the courage to do it”* (p. 311)]. Users with a fixed mindset, on the other hand, were twice as likely to impose self-judgment onto their reflections. They tended to get more frustrated […* I feel very bad and angry* (p. 164)] and condemned by their own failure [*I felt really bad in front of others…* (p. 212)]. Devaluation of the effort and overemphasis on the outcome itself lets users with a fixed mindset occupy themselves with their inadequacies […*I felt very guilty of my incapability and lack of nutrition knowledge* (p. 193)].

##### Mindfulness-vs.-Over-Identification

To be able to express self-compassion, at first one needs to have awareness over her own pain rather than ignoring it. It is important, however, that the awareness is balanced so that the unpleasant experience is not exaggerated and over-identified. The analysis showed that a majority of the users, regardless of their mindset, indeed acknowledged their setbacks […*I thought I was ready to this but was not* (p. 21), *I felt pretty bad about myself at first, but what happened was unavoidable…* (p. 203)]. The difference between mindsets, however, was particularly evident in the way participants emotionally over-identified with their failures. Those with a fixed mindset were twice as likely to perceive their setbacks as more permanent and definitive [e.g., *I feel like I am a loser* (p. 114)], and a disappointing performance was taken as a measure for their lack of worth […*I failed and felt worthless* (p. 237)]. This finding was not as common among those with a growth mindset, implying that the belief that abilities can be cultivated seemed to let users with a growth mindset avoid identifying themselves with their failures and instead allowed recognizing their shortcomings in an objective manner.

##### Common-Humanity-vs.-Isolation

Common humanity is about recognizing that failing and making mistakes are part of the shared human experience that connects us as opposed to causing isolation. While no one in our sample experienced isolation as an outcome of their setback, our findings showed that, albeit less frequently than other two dimensions, few users with a growth mindset recognized their setbacks as vulnerabilities that are shared by all humans, more so than users with a fixed mindset […*Life happens* (p. 126)].

Although participants were reminded of their setback, which can be an unflattering experience, the analysis showed that users with a growth mindset managed to comfort and care for themselves by acknowledging their mistakes mindfully, accepting their humanness and being understanding toward themselves, meanwhile users with a fixed mindset were harsher toward themselves and tend to exaggerate the implications of their mistakes for their self-worth.

## Discussion

Self-tracking technologies ubiquitously measure diverse bodily and behavioral features, store and analyze the accumulated data, and provide numerical feedback, which is received and reflected upon by the user. In the course of reflection, however, numbers lose their objective nature and are converted into subjective knowledge that is susceptible to users' interpretations. This subjective user experience is found to be affected by various parameters such as the type of relationship user builds with the device; while seeing it more as a tutor attribute “advice-giving” role to the numbers, seeing it more as a toy can make the same numerical feedback more playful (32). In the current study, we have addressed user mindsets as one of these parameters and shown that users with different mindsets experience self-tracking differently. Implicit beliefs about the changeability of behavior seem to influence the extent to which users are self-determined toward self-tracking use as well as how they perceive and respond to situations in which their self-tracking goals are not met. For users with a growth mindset, who approach their disappointing performances with more self-compassion, self-tracking data can be perceived as a quest for insight, which is rewarding in its own right. They are characterized by being more open-minded about learning from the data, considering a lack of goal achievement as a starting point for improvement rather than as personal failure. In contrast, those with a fixed mindset appear to be more likely to have external rewards drive their behavior, and perceive the accumulated data as a confirmation of success or failure in reaching a goal.

Our findings also showed that mindsets and the duration of self-tracking use are correlated; the more people believe that behaviors are changeable with the use of self-tracking, the longer they become likely to adopt self-tracking technologies. Discontinuation of self-tracking within shorter periods of time is an increasing concern (33) and may negatively impact the health benefits that self-tracking offers, as these benefits, such as healthy habit formation, are likely to be manifested over longer periods of practice (34). Our findings, therefore, can be illuminating to better understand the underlying mechanisms associated with a higher abandonment rate. Many studies have addressed this issue with a mere focus on the technology itself and interpreted it as a failure of the technology to galvanize users into continuous engagement (35). Our findings, on the other hand, show that independent of the tool, the user mindset may also play a determining role in (dis)continuation of self-tracking use. Accordingly, as people with a fixed mindset tend to be more self-judgmental toward their failure to reach a goal, this could have a significant impact on their overall self-tracking experience, which becomes one of confrontation rather than of learning. Our findings suggest that this may also be a factor in abandoning self-tracking in the longer run, in order to avoid repeated confrontations with negative feedback. It is possible that this motive to avoid repeated confrontations heightens one's mental state of learned helplessness where the user stops trying avoid negative situations after facing them repeatedly, even if the user has the ability to do so (36) and this can lead to discontinuation of self-tracking use within shorter periods of time. That being said, however, it is important to bear in mind that our findings are solely correlational. Hence, another possible interpretation could be that the mindsets users hold are a result of (dis)continuation of self-tracking use. After all, mindsets can change over time and a growth mindset can be taught through new experiences and interventions (9). It is possible that experiencing the benefits of self-tracking after long-term engagement, even if in a rather smaller scale, i.e., heightened self-awareness, can encourage people to believe that behavior change with self-tacking is possible and this belief can let users lean toward adopting a more growth mindset. More experimental research using specific mindset interventions can assist in to further tackling the causality of this link.

### Mindset and Its Implications for Self-Tracking Use

Overall, the findings of this study indicate that different mindsets users hold could affect how self-determined users are toward the use of self-tracking, that is, how tolerant vs. self-judgmental they become toward their own self-tracking performance, and how long they will continue to be engaged with the self-tracking practice. In the light of these findings, the current study makes a number of contributions to personal informatics research and has implications to better understand the user and the interaction a user has with the system itself.

The nature of self-tracking measurements and the way it might affect users' understanding of accomplishment have become a topic of interest in recent personal informatics literature (37, 38). A field study on pedometers shows that the presence vs. absence of numerical feedback (i.e., number of step counts) leads to different self-talks among users (39). In the feedback condition, users tended to be more self-critical when they walked less and approached walking itself more as a task that needs to be accomplished compared to those who did not receive in-the-moment feedback on their steps. This negative self-talk and performance focus in the presence of numerical feedback seems to point out the direction that numerical feedback is perceived as a definitive judgment of their walking abilities. In light of our current findings where mindset has been found to be related to influence the interpretation of data, it is possible that the effects of negative self-talk are more pronounced for people with fixed mindset. Conversely, because mindsets are known to be impacted by external factors or interventions, such as information about brain plasticity, or mastery-oriented task framing, self-tracking data may play a role in transforming users' implicit beliefs about their abilities toward a more fixed direction.

Furthermore, because users' mindsets play an important role in the way self-tracking performance is being evaluated, it affects the dynamics of the relationship the user builds with the self-tracking tool. As outcome is the most important for people with a fixed mindset, it has been shown that their goal mostly becomes to “*look smart rather than to become smart*” (40). They are also driven by labels, either to receive positive labels or to avoid negative labels, even if it includes deception. This motivation can be so strong among people with a fixed mindset that they are indeed found to have a higher tendency to cheat in order to avoid from negative labels (41). This becomes highly relevant in understanding self-tracking users with fixed mindsets. The measurement nature of self-tracking paves the way for labeling oneself, reaching vs. not reaching the numbers one has set for oneself can easily be translated into succeeding vs. not, defining users' progress and accomplishments by the numbers. Correspondingly, one's progress or lack of it becomes the object of constant examination by the user herself. Combination of heightened focus on the outcome together with avoidance of negative labeling can prompt users, especially those with a fixed mindset, to perceive the monitored data as personal representation of the self and make them susceptible to deception. For example, it has been reported that some users “cheat” the system (e.g., shaking a step counter) and optimize the tracked parameter rather than the underlying target behavior (42). This desire to meet the numerical goal can be a driving force for someone with a fixed mindset who is afraid to get labeled as a failure.

### Limitations

This study is not without its limitations. First, in the current study, user mindsets were identified based on participants' self-report scores on the mindset scale. While this approach has allowed us to establish mindset as a continuous variable and explore its role in the self-tracking context for the first time, we did not have interventions in the first place that would actively impact a person's mindset, allowing us to experimentally control mindset, at least to an extent. Please take note that we agree that self-report is a primary way of measuring mindset and is not a limitation in and of itself. Instead, the absence of explicit manipulation in this particular study has been pointed out as a limitation as such manipulation would have allowed for stronger causal inferences. Having used self-report scores, our research was correlational and exploratory in nature, providing a basis for more specific causal predictions and hypothesis testing in future studies.

The measure for mindset turned out to consist of multiple components, as shown by the principal component analysis. While all items loaded high on the first component, an interesting distinction between the positively framed items and the negatively framed ones became visible in the second component. Even though we did recode the negatively framed items to be coded in the same direction as all other items, this framing in itself could have been the reason for the second component to show up in our data. We have decided to combine all items into one construct, but we agree with (4, 5) that further research is needed to fully understand the workings of the concept.

Additionally, while the emphasis of the mindset scale is on progress and improvement, one might argue that few items also partially lay stress on the outcome, tapping more into measuring self-efficacy than the growth mindset. We see self-efficacy as the perceived ability to succeed, and growth mindset as the belief that the behavior can be improved, regardless of the outcome. As these concepts are conceptually close yet distinct, we expect them to correlate. Future studies can further explore this relationship between self-efficacy and growth mindset in the self-tracking context.

Our participant pool consisted of people who had been voluntarily self-tracking. This limits the variability in levels of self-determination and may impact the generalizability of our findings. Recruiting participants with varying levels of self-determination (e.g., people who start practicing self-tracking after receiving a self-tracking tool as a gift or upon doctor prescription) can further aid in analyzing the relationship between mindset and self-determination in the self-tracking context for a wider population.

Lastly, while the current study used a qualitative approach providing richness and granularity in understanding the ways in which users react to a setback, we did not obtain the actual tracking data to compare our self-report results against. Acquiring the monitored data can enrich the interpretation and help to better understand the self-tracking experiences across users with different mindsets, as well as across different levels of actual exercise behavior. Despite these limitations, however, the findings of the current study shed light on individual differences in self-tracking experiences with its novel focus on addressing users' mindsets.

### Design Considerations

Understanding of mindsets shows that users vary in the ways in which they interpret and respond to feedback about their abilities; those with a growth mindset use it to learn and improve their skills while those with a fixed mindset use it as an affirmation of talent. Despite having users with varying expectations, however, self-tracking devices are designed to construe the behavior and deliver the feedback in a certain one size fit all format that is quantified. Such formatting makes the self-tracked behavior subject to measurement and hence susceptible to analysis, comparison, and, for some cases, competition and nudging its users toward a more fixed mindset about their abilities. Designing self-tracking technologies with a mastery orientation in mind can help to steer users' focus away from a fixation on the outcome itself and help to deliver a more meaningful and long-term engagement with the device, the process and experience of exercise, and eventually, one's own body. We argue that to do so, we need to highlight the experience that self-tracking offers over numbers by means of (a) reconstructing the definition of exercise, (b) providing a guided reflection on the learning experience, and (c) recognizing/utilizing self-kindness as a reflective feature.

#### Reconstruct the Definition of Exercise

Describing exercise solely as a portrayal of monitored data and disregarding other experiences it delivers simply because they are not quantifiable (e.g., the enjoyment one gets from exercising) can prompt users to treat exercise as a prescription of some sort (e.g., if you want to pursue a healthier lifestyle, you need to take 10,000 steps every day) whereby, when performed just at the right level, the body can be “fixed” (43). To reconstruct this experience, we need self-tracking technologies that allow setting up different goals that are more oriented toward mastery and open-minded curiosity than toward performance or proving oneself (e.g., What would you want to learn today?). Prompting a growth mindset can allow users to pose the question “what is a healthy level of exercise for me?” rather than asking “what is the level of exercise people would expect from me?” In addition, a holistic appreciation of exercise can be stimulated where performance is not the ultimate goal, but rather a broader set of embodied and contextualized experiences associated with exercise. Providing games that take away focus from numbers, for example, can create opportunities for users to spend the time enjoying the surrounding environment (e.g., a Tree Collection game: Have you seen any trees on your running route today? Can you name their species?).

#### Guided Reflection on the Learning Experience

To prompt users toward a mastery mindset, self-tracking technologies need to reframe the notions of failure and success in the context of exercise, irrespective of the outcome. Self-tracking technologies have a tendency to focus positive feedback on the ability to reach one's goal, and not appreciate the effort. Similar to the findings where students are found to be less likely to accept a new challenge when praised for their ability instead of effort (8), it is possible that users of self-trackers will demonstrate an avoidance motivation, that is, they will fear exposing their incompetence, and will thus avoid challenging themselves. Learning with relation to effort and not merely the outcome, however, can aid in adopting a learning orientation (e.g., heart rate as an indicator of effort, instead of miles or steps). Also, notions such as endurance and “pleasurable pain” should be acknowledged and embraced as they signify the control and exploration of new bodily boundaries.

#### Self-Kindness as a Reflective Feature

Self-tracking technologies should create enough room for users to show self-kindness. They should provide feedback that allows users to savor their bodies' vitality and resilience, without giving in to illusions of bodily perfection, and to guide them to be self-compassionate and self-caring without giving in to narcissism. In a similar vein, acquiring a mindful state and acknowledging what is happening in your mind as you exercise (e.g., feeling your body move) can also be a way of showing self-compassion, which helps to reconceptualize exercise.

### Conclusion

The present study explores the effects of users' mindsets on their self-tracking experience with regard to exercise behavior. Our findings suggest a connection between a person's mindset and the way they process information feedback provided by self-tracking devices. Mindset impacts the focus that people have while exercising, and leads to distinct differences in dealing with the experience of reaching or not being able to reach one's goals. People with a growth mindset tend to show more self-compassion and are less self-judgmental toward themselves when failing to reach a goal, than people with a fixed mindset. Also, our findings suggest that people with a growth mindset are more likely to continue with self-tracking practices over long periods of time. Whilst the reliability and generalizability of the present study need further large-scale studies to validate the findings, this study presents one of the first empirical works that explore the role of mindset as an important determinant in shaping the self-tracking experience. Considering the fact that users' responses to self-tracking data is one of the core dimensions of self-tracking practice, we believe our research contributes to an improved understanding of the psychology of self-tracking, while at the same time providing avenues for design improvements of self-tracking devices and apps.

## Data Availability Statement

The raw data supporting the conclusions of this article will be made available by the authors, without undue reservation.

## Ethics Statement

The studies involving human participants were reviewed and approved by Ethical Review Board of the Human-Technology Interaction group at Eindhoven University of Technology. The patients/participants provided their written informed consent to participate in this study.

## Author Contributions

EH performed the statistical analysis and wrote the first draft of the manuscript. PR, JL, and WI reviewed the sections of the manuscript. All authors contributed to conception and design of the study, manuscript revision, read, and approved the submitted version.

## Conflict of Interest

The authors declare that the research was conducted in the absence of any commercial or financial relationships that could be construed as a potential conflict of interest.

## Publisher's Note

All claims expressed in this article are solely those of the authors and do not necessarily represent those of their affiliated organizations, or those of the publisher, the editors and the reviewers. Any product that may be evaluated in this article, or claim that may be made by its manufacturer, is not guaranteed or endorsed by the publisher.

## Appendix A. Mindset scale: adjusted from the Revised Implicit Theories of Intelligence (self theory) Scale

1 To be honest, I do not think I can really change my behavior with a self-tracking device.
2 I can make small changes, but I do not have the ability to completely change my behavior.
3 Feedback from my tracker has solely the purpose of judging me.
4 I do not like failure, because it shows my lack of ability.
5 When I keep applying effort over time, I think I can improve my behavior with the use of my tracker.
6 With the feedback of my tracker, I can manage to improve my behavior.
7 My behavior is flexible, I can change it with the use of my tracker.
8 I believe I have the ability to change my behavior considerably over time.

## Appendix B. Self-Compassion Scale-Short Form (SCS-SF)

1 I try to be understanding and patient toward those aspects of my personality I do not like.
2 When I am going through a very hard time, I give myself the caring and tenderness I need.
3 I try to see my failings as part of the human condition.
4 When I feel inadequate in some way, I try to remind myself that feelings of inadequacy are shared by most people.
5 When I experience an event of failure, I try to take a balanced view of the situation.
6 When something upsets me, I try to keep my emotions in balance.
7 I am disapproving and judgmental about my own flaws and inadequacies.
8 I am intolerant and impatient toward those aspects of my personality I do not like.
9 When I am feeling down, I tend to feel like most other people are probably happier than I am.
10 When I fail at something that is important to me, I tend to feel alone in my failure.
11 When I fail at something important to me, I become consumed by feelings of inadequacy.
12 When I am feeling down I tend to obsess and fixate on everything that is wrong.

## Appendix C. Motivation for self-tracking scale: adjusted from the Revised Sport Motivation Scale

1 Because it gives me pleasure to learn more about myself.
2 Because it is very interesting to learn how I can improve myself.
3 Because self-tracking reflects the essence of whom I am.
4 Because self-tracking is an integral part of my life.
5 Because I have chosen self-tracking as a way to develop myself.
6 Because I found it is a good way to develop aspects of myself that I value.
7 Because I would feel bad about myself if I did not take the time to do self-tracking.
8 Because I feel better about myself when I do self-tracking.
9 Because people I care about would be upset with me if I did not do self-tracking.
10 Because I think others would disapprove me if I did not do self-tracking.
